# Identification of algal rich microbial blooms in the Sellafield Pile Fuel Storage Pond and the application of ultrasonic treatment to control the formation of blooms

**DOI:** 10.3389/fmicb.2023.1261801

**Published:** 2023-10-04

**Authors:** Lynn Foster, Christopher Boothman, Scott Harrison, Peter Jenkinson, Jon K. Pittman, Jonathan R. Lloyd

**Affiliations:** ^1^Research Centre for Radwaste Disposal and Williamson Research Centre for Molecular Environmental Science, Department of Earth and Environmental Sciences, The University of Manchester, Manchester, United Kingdom; ^2^National Nuclear Laboratory, Central Laboratory, Sellafield, Seascale, United Kingdom; ^3^Sellafield Ltd., Seascale, United Kingdom

**Keywords:** Chrysophyceae, Pile Fuel Storage Pond, spent nuclear fuel pond, algae bloom, ultrasonic treatment

## Abstract

The presence of microorganisms in a range of nuclear facilities has been known for many years. In this study the microbial community inhabiting the Pile Fuel Storage Pond (PFSP), which is a legacy open-aired facility on the Sellafield nuclear site, Cumbria, UK, was determined to help target microbial bloom management strategies in this facility. The PFSP is currently undergoing decommissioning and the development of prolonged dense microbial blooms reduces the visibility within the water. Such impairment in the pond water visibility can lead to delays in pond operations, which also has financial implications. Efforts to control the microbial population within the PFSP are ongoing, with the installation of ultrasonic treatment units. Here next generation sequencing techniques focussing on broad targets for both eukaryotic and prokaryotic organisms were used to identify the microbial community. On-site monitoring of photosynthetic pigments indicated when microbial blooms formed and that eukaryotic algae were most likely to be responsible for these events. The sequencing data suggested that the blooms were dominated by members of the class Chrysophyceae, a group of golden algae, while evidence of cyanobacteria and other photosynthetic bacteria was limited, further supporting eukaryotic organisms causing the blooms. The results of sequencing data from 2018 was used to inform a change in the operational settings of the ultrasonic units, while monitoring of the microbial community and photosynthetic pigments trends was extended. Since the changes were made to the ultrasonic treatment, the visibility in the pond was significantly improved, with an absence of a spring bloom in 2020 and an overall reduction in the number of days lost due to microbial blooms annually. This work extends our knowledge of the diversity of microbes able to colonise nuclear fuel storage ponds, and also suggests that sequencing data can help to optimise the performance of ultrasonic treatments, to control algal proliferation in the PFSP facility and other inhospitable engineered systems.

## Introduction

The continued development and application of DNA-based culture-independent techniques, has increased our understanding of the diversity of microbial life, and extreme environmental conditions within which microorganisms can live (Blanco-Rivero et al., 2005; Pikuta et al., 2007). Hostile radioactive environments are known to be colonised by a variety of microorganisms, and laboratory tests have shown that some are capable of withstanding doses of radiation significantly higher to anything seen in natural environments on Earth (Daly et al., 2004; Daly, 2009; Rivasseau et al., 2010). The presence of microorganisms in spent nuclear fuel ponds (SNFPs), and other similar nuclear facilities has been documented for many years (Sarró et al., 2003; Chicote et al., 2005; Rivasseau et al., 2010; Dekker et al., 2014; Karley et al., 2017; Bagwell et al., 2018; Silva et al., 2018). Both culture-dependent, and independent techniques have been utilised to assess the microbial communities that colonise these radioactive environments (Chicote et al., 2005; Sarró et al., 2007; Bagwell et al., 2018). The colonisation of SNFPs by microorganisms has proved to be problematic for a number of reasons including, biofouling of infrastructure of the facilities (Gadd, 1990; Barton et al., 2022), and the potential for microbial-induced corrosion. Perhaps most problematic for outdoor facilities are photosynthetic microbial blooms, which reduce visibility within the pond water, and hence inhibit pond operations and decommissioning activities (Ashworth et al., 2018; MeGraw et al., 2018; Foster et al., 2020a).

Two open-air legacy SNFPs on the Sellafield nuclear processing site in Cumbria, UK have reported disruptive microbial blooms, and the application of next generation sequencing techniques has determined the organisms responsible for them (MeGraw et al., 2018; Foster et al., 2020a). Prevailing conditions within each of the SNFPs influence which bloom forming organisms dominate. For example, in a circum-neutral pH SNFP, blooms were dominated by *Haematococcus* sp. (MeGraw et al., 2018), a eukaryotic alga that produces the red carotenoid astaxanthin, which is known to have antioxidant properties (Liu et al., 2010), and which likely provides protection against the radioactivity in the pond (which can lead to the formation of toxic free radicals). Conversely, in the First Generation Magnox Storage Pond (FGMSP) where the pH is maintained at ∼11.4, a cyanobacterium belonging to the genus *Pseudanabaena* was identified as being a key bloom forming organism (Foster et al., 2020a). Laboratory tests indicated that a close relative of the *Pseudanabaena* sp. identified in the pond produced excess polysaccharides when x-irradiated that could serve as a defence mechanism to withstand high doses of radiation (total dose of 95 Gy) (Foster et al., 2020c).

The Pile Fuel Storage Pond (PFSP) is another legacy SNFP on the Sellafield site. Constructed in the late 1940s, the PFSP was the first SNFP to be built on the Sellafield site, and it became operational from the 1950s (Carlisle, 2013; Ayoola, 2018). The PFSP has had a varied operational history including the cooling and decanning of Windscale fuel, receipt of a range of miscellaneous intermediate waste, and fuel contaminated items (Carlisle, 2013; Sellafield, 2019). All active operations ceased in the pond in the 1970s, and the facility was placed in passive care and management (International Atomic Energy Agency, 2015; Ayoola, 2018). The PFSP is currently undergoing decommissioning, which has already resulted in the removal of significant quantities of the radioactive materials and waste (Sellafield, 2019; Nuclear decommissioning authority and Sellafield, 2021).

The PFSP is an open-air facility and is therefore subject not only to the ingress of rain but also to environmental debris such as bird guano, which provides a source of carbon and nitrogen. Like other open aired SNFPs on the Sellafield site, the PFSP is colonised by microorganisms (Nuclear decommissioning authority and Sellafield, 2016) that form dense blooms. Prior to 2018, Sellafield reported microbial bloom episodes that lasted for several months and significantly reduced the visibility within the pond water resulting in the inability to carry out various operations (Effluent Team Sellafield Ltd, Personal correspondence). The planned decommissioning schedule for the PFSP (and the associated costs) are significantly impacted by down time associated with the prolonged periods of reduced in-pond visibility caused by algal blooms. Before the commencement of this study, there was no information about the identity of the microorganisms in the PFSP, or whether the microbial blooms were caused by eukaryotic algae or cyanobacteria, or a combination of both. The strategies available to control the microbial populations in the PFSP are limited due to constraints imposed by the pond infrastructure and the nature of the stored materials. Sellafield Ltd have therefore deployed ultrasonic units into the PFSP in an attempt to control the microbial populations, as this commercial off-the-shelf technology was deemed the easiest to deploy given the infrastructural constraints associated with the facility, and would have no adverse impact on the water chemistry or the stored inventory. Ultrasonic waves at frequencies above 20 kHz often cause the phenomenon of acoustic cavitation in a liquid medium leading to the formation of high energies, temperatures and pressures at a microscale, which can break up cell aggregates, inhibit cell buoyancy causing sedimentation, and can cause damage and destruction to microbial cells (Broekman et al., 2010). Use of ultrasound at specific frequency, power and duration settings is therefore considered to be an efficient method to control microbial blooms (Dehghani, 2016). The LG Sonic ultrasonic units, deployed in the PFSP, operate using low-power ultrasound, which does not result in acoustic cavitation but rather restricts cells rising though the water column into the photosynthetic active region of the water body (LG Sonic, 2023).

The aim of this study was to examine the microbial community in the PFSP between 2018 and 2020, a period during which several algal blooms were recorded, both before and after deployment and programming of the ultrasonic units. This was in order to characterise the community structure of this pond for the first time and validate a bloom control strategy. Pond water samples were obtained for examination using next generation sequencing, in order to provide information on the whole microbial community present. Sequencing results from the 2018 samples were also used to inform appropriate programme selection for the ultrasonic units, implemented in April 2019. Insight from this study will benefit the development of algal bloom control strategies at the PFSP facility as well as other equivalent engineered pond sites.

## Materials and methods

### Safety statement

The PFSP is situated on a nuclear licenced site, as such the acquisition and handling of samples collected from this facility requires the adherence to strict safety protocols. The samples collected from the pond contain radionuclides and were handled by Suitably Qualified and Experienced Personnel (SQEP). All samples were subject to rigorous monitoring procedures in designated radioactive laboratories prior to any experimental work or transportation.

### Description of the Pile Fuel Storage Pond (PFSP), sample collection, and pond conditions

The PFSP had been operating under a state of passive maintenance and care since the 1970s, and as such, is subjected to routine checks to ensure the conditions within the facility remain stable. However, such checks do not include measurements such as PO_4_^3–^, NO_3_^–^, or total organic carbon levels, and as such these parameters were not available for this study. The facility has a pH that typically ranges between 9.2 and 9.5, however this can reach pH ∼10 during the bloom periods. Over time, there has been a build-up of radioactive sludge on the floor of the pond, made up of corroded fuel products, biomass such as bacteria and algae, and various other debris (Nuclear decommissioning authority and Sellafield, 2016). The PFSP is not purged, however, the pond water level is closely managed, which necessitates the discharge of water from the pond to accommodate rainwater. The pond consists of two sub-ponds that are divided by a central wall that is open at the centre point, and therefore the water within the two sub-ponds is hydraulically linked. A local effluent treatment plant (LETP) was installed in the PFSP to manage the levels of soluble radioactivity in the pond water via an ion exchange process and to remove radioactive solid particles via a sand bed filter (International Atomic Energy Agency, 2015). The predominant radioisotopes within the PFSP are ^137^Cs and ^90^Sr, which account for ∼99% of the total soluble activity (International Atomic Energy Agency, 2015). Activity concentrations of ^137^Cs and ^90^Sr recorded during the study period were 191–289 Bq mL^–1^ and 140–148 Bq mL^–1^, respectively, resulting in gamma dose rates that were typically below 20 μSv h^–1^.

Between March 2018 and August 2020, PFSP water samples were obtained on nine occasions (Supplementary Table 1). On four of these, algal blooms were evident during sampling (March 2018, April 2018, September 2018, and March 2019), whilst for the remainder, no algal blooms were evident. All pond water samples (volumes ranging from 384 to 996 ml) were obtained by Sellafield Ltd personnel from a depth of 1 m using a hose and hand operated vacuum pump which dispensed the pond water into sterile containers, from the same sampling location.

The abundance of photosynthetic organisms was monitored in the ponds using the EXO total algae sensor (WTW Wissenschaftlich^®^ Technische Werkstätten, Germany). This is a dual-channel fluorescence sensor that provides estimations of the concentration of the photosynthetic pigments chlorophyll *a* (Chl *a*) and phycocyanin, which are used as a proxy for the abundance of a broad range of photosynthetic organisms and cyanobacteria, respectively. The Chl *a* molecule is quantified using a blue excitation beam (470 ± 15 nm) emitted by the sensor, whilst the phycocyanin molecule is quantified by an orange excitation beam (590 ± 15 nm). The temperature of the pond was recorded throughout the sampling period and showed seasonal variations with lows of ∼3.8°C in January and highs of ∼20°C in August (Supplementary Table 1).

Two ultrasonic E-line algae control units (LG Sonic HQ, Netherlands) were deployed in the PFSP on the 6^th^ April 2018. One unit was deployed to mitigate the risk of algal bloom development in the East sub-pond, while the second was deployed to mitigate the risk of bloom development on the West sub-pond. Both units are able to be set to any one of 12 programmes, each with a different range of ultrasonic parameters which include; frequency, amplitude, waveform, and signal duration to control different types of algae (LG Sonic, 2023). Following initial deployment, Sellafield Ltd recognised that the most appropriate programme selection to optimise effectiveness would not be possible until sequencing data obtained from this study was available. As such, during the initial deployment period, Sellafield Ltd ran through three programme selections, each for a period of at least 4 weeks, to help identify a programme that was appropriate to control the microbial communities in the pond (without sequencing data). On 8^th^ April 2019 the 16S and 18S rRNA gene sequencing data was used to inform a further change to the ultrasonic programme selection, which was maintained for the remainder of the study. During the course of the study both ultrasonic units were operating on the same settings as each other.

### DNA extractions

The pond water samples were transferred to the National Nuclear Laboratory (NNL) Central Laboratory (Cumbria, UK), where they were stored at 4°C prior to further radiological assessments and DNA extraction. Since the samples were radioactive, the DNA extractions were carried in a dedicated laboratory for handling radioactive samples. All samples were handled in a Grant Bio UVC/T-M-AR DNA/RNA UV cleaner box (Grant Bio, Cambridge, UK), where the water was filtered through a 0.2 μm sterile filter using a vacuum pump. DNA extractions were carried out using the Qiagen DNeasy Powerwater Kit (Qiagen, Germantown, MD, USA) following the manual provided within the kit. DNA was eluted to a final volume of 100 μL, and checks were made to ensure there was no remaining radioactivity associated with the DNA samples. Extracted DNA samples were stored at −20°C prior to being transported to the University of Manchester (UoM). Upon receipt of the samples at the UoM, the samples were once again checked for any radioactivity, and were stored at −20°C.

### Sequencing and analysis of the 16S and 18S rRNA genes

The DNA samples collected during the study were sequenced using the Illumina MiSeq platform (Illumina, San Diego, CA, USA), to determine the prokaryotic and eukaryotic microbial communities through the analysis of the 16S and 18S rRNA genes, respectively. The protocol used for the sequencing of both rRNA genes and the downstream analysis of the sequencing data, followed that outlined by Foster et al. (2020a). Roche High Fidelity PCR system (Roche Diagnostics Ltd., Burgess Hill, UK) was used to carry out the PCR amplifications in 50 μL reactions, using the specific conditions required for the different sets of primers (Supplementary Table 2). All PCR products were purified and normalised to ∼20 μg, each using the SequalPrep Normalization Kit (Fisher Scientific, Loughborough, UK), before being pooled in equimolar ratios. The 16S rRNA gene sequencing was performed with a 4 pM PhiX spiked library following the protocol outlined by Kozich et al. (2013), whilst the 18S rRNA gene sequencing run was performed using a 9 pM spiked PhiX library. The addition of PhiX, which is a well characterised phage genome, allows for calibrations and quality controls during the sequencing process. Sequencing pipelines were used to divide the raw 16S and 18S rRNA gene sequences into sample barcodes (up to one mismatch permitted). Steps within the pipeline included quality control and trimming using Cutadapt (Martin, 2011), FastQC (Billi and Potts, 2002), and Sickle (Joshi and Fass, 2011), and MiSeq error corrections were made using SPADes (Nurk et al., 2013). Pandaseq (Masella et al., 2012) allowed the forward and reverse reads to be incorporated into full-length sequences, and any chimaeras present were removed using ChimeraSlayer (Haas et al., 2011). UPARSE (Edgar, 2013) was used to generate 16S rRNA gene operational taxonomic units (OTUs) that were further classified by Usearch (Edgar, 2010) at the 97% similarity level, additionally singletons were removed. For the 18S rRNA gene sequencing data the OTUs were generated and classified using VSEARCH (Rognes et al., 2016). The original OTUs detected in Qiime (Caporaso et al., 2010) were used to conduct rarefaction analysis for both 16S and 18S rRNA gene sequencing data. Taxonomic assignment of the 16S rRNA gene data was carried out using the RDP classifier V2.2 (Wang et al., 2007), UCLUST performed the 18S rRNA gene taxonomic assignments using the Silva119 database (Quast et al., 2013). Diversity measurements to compare pond samples were carried out using the Shannon Wiener and Simpson Diversity indexes.

### Estimating the average copy number of the 16S and 18S rRNA genes using quantitative polymerase chain reaction (qPCR)

The Aria MX qPCR machine with Aria1.8 software (Aligent, Santa Clara, CA, USA) was used to carry out the quantification of the 16S and 18S rRNA genes. A mastermix of qPCR reagents (Supplementary Table 3) were prepared and 23 μL aliquots dispensed in the polypropylene 96-well plates used to perform the qPCR runs, the reactions were made up to 25 μL with the addition of the sample DNA. The V1–V3 region of the 16S rRNA gene was targetted by the 8F and 519R primers (Lane, 1991), whilst the V9 region of the 18S rRNA gene was targetted by the 1391F and EukBr primers (Tanaka et al., 2014). A 10-fold dilution series of standards were used during each run, and each sample was run in triplicate. Thermal cycling conditions appropriate for each set of primers were used (Supplementary Table 4), and after 35 cycles a dissociation curve was made for the qPCR products by increasing from the annealing temperatures to 94°C, with a ramp rate of 0.01°C per second. AriaMX v1.8 software (Aligent, Santa Clara, CA, USA) was used to carry out the baseline and threshold calculations, and generate the standard curves (Supplementary Figures 1, 2). The absolute quantification by the standard-curve (SC) method (Brankatschk et al., 2012) was then used to quantify the concentration of the target genes. All qPCR results include standard deviations.

## Results and discussion

### Analysis of photosynthetic pigments in the Pile Fuel Storage Pond

An EXO total algae sensor was installed in the Pile Fuel Storage Pond (PFSP) to monitor the growth of photosynthetic bloom forming microorganisms. The dual sensor detects the fluorescence of the photosynthetic pigments chlorophyll *a* (Chl *a*) and phycocyanin, which are indicative of a broad range of photosynthetic organisms and cyanobacteria, respectively. Throughout the study period (25^th^ January 2018 to 20^th^ October 2020), the concentration of Chl *a* (0.66–46.4 μg L^–1^) was consistently higher than that recorded for phycocyanin (0.07–1.94 μg L^–1^), and significantly higher during bloom periods (Supplementary Figure 3), providing evidence that cyanobacteria were unlikely to be the main bloom forming organisms during this period. Operators at the Sellafield facility reported that where the concentration of Chl *a* exceeded 10 μg L^–1^, there was a reduction in the visibility within the PFSP, and that this was considered to be caused by a microbial bloom (Effluent team, Sellafield Ltd, Personal correspondence). Furthermore, where the concentration of Chl *a* exceeded 20 μg L^–1^, there was a complete loss of visibility within the PFSP which resulted in all operations including waste retrievals in the pond being halted.

### Chlorophyll *a* concentration trends between 25^th^ January 2018 and 20^th^ October 2020 showed multiple bloom periods, with a reduction in number of days where visibility was affected year on year

**FIGURE 1:**
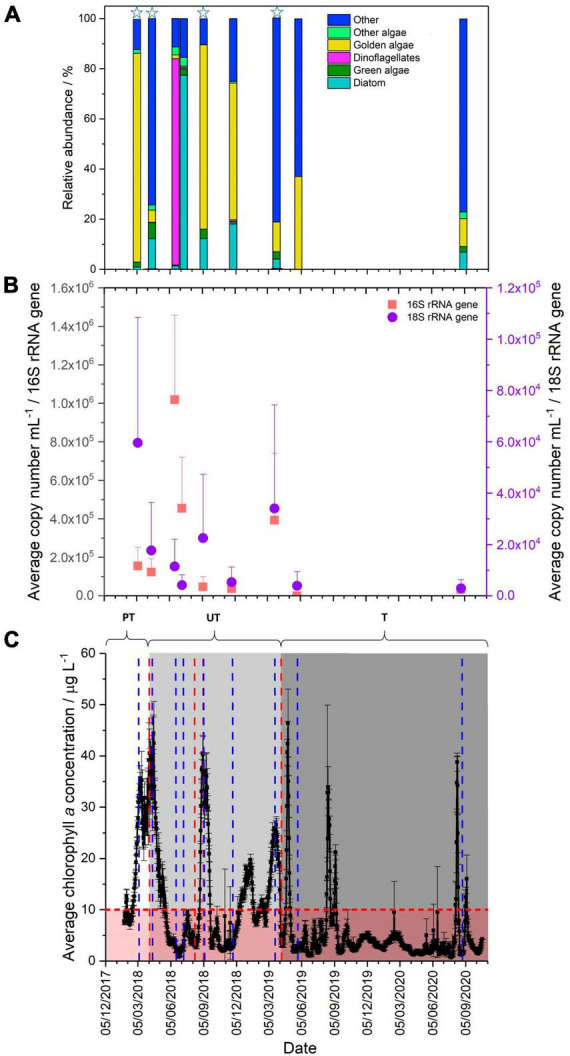
**(A)** Comparison of eukaryotic community present in the Pile Fuel Storage Pond, grouped to highlight the relative abundance of photosynthetic organisms, stars highlight bloom samples (*Chl a* concentrations >10 μg L^–1^). **(B)** qPCR quantification of the 16S (red) and 18S (purple) rRNA gene copy number mL^–1^ (error bars denote the standard deviation). **(C)** Average chlorophyll *a* concentrations (μg L^–1^) collected between 25/01/2018-20/10/2020. PT, pre-treatment of ultrasonic frequencies; UT, untargeted (light grey panel), ultrasonic units operating on non-specific settings; T, targetted (grey panel), ultrasonic units operating on settings specified by LG Sonic. Error bars denote standard deviation, the horizontal red line marks threshold above which visibility becomes compromised, the red panel represents good visibility in the pond, and the blue lines indicate time points where samples were collected, vertical red lines denote when the ultrasonic settings were changed.

In 2018, two algal bloom events were recorded in the PFSP during which Chl *a* concentrations exceeded 10 μg L^–1^ for 142 days and exceeded 20 μg L^–1^ for 82 days (41% and 25% of the total recorded days, respectively). The first bloom formed in February 2018, and did not subside until the end of April 2018. During this spring bloom, there were two peaks where the maximum Chl *a* concentrations were 35.6, and 44.4 μg L^–1^ (Figure 1C). A shorter bloom was recorded at the end of August 2018 (late summer bloom) that lasted approximately 1 month with a maximum Chl *a* concentration of 40.5 μg L^–1^.

Two further blooms were reported in 2019, however, Chl *a* concentrations exceeded 10 μg L^–1^ for 109 days (∼28% of the total days) and exceeded 20 μg L^–1^ for 34 days (∼9%). The two blooms observed in 2019, occurred at similar times to those observed in 2018, namely March-April (spring bloom) and August-September (late summer bloom). The maximum concentration of Chl *a* recorded in the March–April spring bloom in 2019 was lower than that observed in 2018, at 26.6 μg L^–1^, whilst the maximum Chl *a* concentration in August was 32.9 μg L^–1^. In 2020, the spring bloom that had been observed in previous years did not form, and there were only 11 days where the concentration of Chl *a* exceeded 10 μg L^–1^, and only 5 days where it exceeded 20 μg L^–1^. There was only one bloom recorded, which occurred in August and although it was of short duration, the Chl *a* concentrations peaked at 38.8 μg L^–1^.

### Estimation of the average copy number mL^–1^ of the 16S and 18S rRNA gene using the quantitative polymerase chain reaction (qPCR)

Quantitative polymerase chain reaction targeting of 16S and 18S rRNA genes was used to determine the relative abundance of prokaryotic and eukaryotic organisms in the PFSP. The results for the 16S rRNA genes showed variation in the overall copy number during the sampling period, ranging from 10^1^ to 10^6^ mL^–1^ (Figure 1B). During the spring bloom in 2018, the samples taken in March and April had lower copy numbers mL^–1^ (1.6 × 10^5^ mL^–1^ and 1.2 × 10^5^mL^–1^, respectively) than in the subsequent samples collected in June (1 × 10^6^mL^–1^) and July (4.5 × 10^5^mL^–1^), when the bloom had receded and visibility within the pond had been restored. The increased abundance of prokaryotic organisms in June and July was not sufficient to influence visibility, indicating that during this sampling campaign, prokaryotic organisms were not the primary cause for the loss in visibility reported in the PFSP. The March 2019 bloom sample showed an elevated average copy number of 3.9 × 10^5^ mL^–1^. Although this was much higher than in some of the other background water samples, it did not exceed the abundance observed in June 2018. Comparisons between the copy number mL^–1^ recorded for the blooms samples compared to non-bloom samples did not show any significant difference when analysed by one-way ANOVA (*P*-value 0.379, F crit 4.24), providing further evidence that prokaryotic organisms were not responsible for the blooms. Given that the abundance of prokaryotic organisms was lower than that recorded when visibility was reported to be good (i.e., no bloom evident), it is unlikely that prokaryotic organisms were affecting the visibility of the pond in March 2019.

Quantification of the relative abundance of eukaryotic organisms indicated that the average copy number mL^–1^ of the 18S rRNA gene increased during bloom periods, when pond water Chl *a* concentrations were high. For samples taken during bloom periods in (March, April and September 2018, and March 2019), average copy numbers mL^–1^ ranged between 1.7–6.0 × 10^4^mL^–1^. This compares to average copy numbers mL^–1^ of 2.8–5.3 × 10^3^ mL^–1^ for samples obtained when no bloom was evident in the pond. The copy number mL^–1^ for bloom samples were significantly different to those recorded for non-bloom samples (one-way ANOVA, *P*-value 0.008, F crit 4.24), which provides further evidence that changes in the abundance of eukaryotic organisms were contributing to the reduced visibility reported in the PFSP. The patterns in the copy numbers mL^–1^ observed for the 16S and 18S rRNA genes suggest that it is the relative abundance of eukaryotic organisms that impacts the visibility within the PFSP.

### Eukaryotic sequencing

**FIGURE 2:**
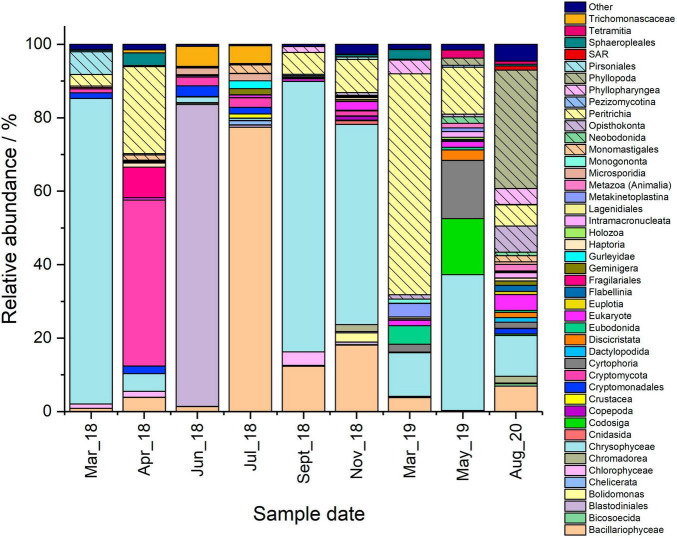
Microbial community comparison of the Pile Fuel Storage Pond based on the 18S rRNA gene sequencing data. Samples were collected between March 2018 and August 2020. Taxa accounting for 1% or less of the combined abundance were omitted and placed within the other group.

The main taxa identity of the eukaryotic community that inhabited the PFSP between March 2018, and August 2020 was determined through the analysis of 18S rRNA gene sequencing data. In total, there were four samples collected during microbial blooms where Chl *a* concentrations were in excess of 20 μg L^–1^. A further five samples were collected when there was good visibility in the pond, and Chl *a* concentrations were below 3.5 μg L^–1^. Whilst there were variations seen in the total number of operational taxonomic units (OTUs) identified across the samples (82–184 OTUs), there was no significant difference in the diversity recorded between bloom, and non-bloom samples when analysed by the Shannon Wiener Index, and Simpson Diversity. The 18S rRNA gene sequencing data revealed several taxa that were identified in all the samples analysed (Figures 1A, 2), these were; the golden alga class Chrysophyceae (0.6–83.2%), the green alga class Chlorophyceae (0.1–6.4%), the diatom class Bacillariophyceae (<0.1–77.4%), the clade Cryptomycota (<0.1–45.2%), and Opisthokonta, which is a broad grouping of eukaryotes (<0.1–7.1%). The subclass of ciliates Peritrichia were also identified in most of the samples (<0.1–60.1%), but were not present in the June-18 sample.

There were four samples collected that captured three out of the six blooms that occurred during the sampling campaign. Of interest was the presence of OTUs affiliated with algal taxa, since rises in Chl *a* concentrations coincided with elevated copy numbers mL^–1^ of the 18S rRNA gene, suggesting such organisms were responsible for the formation of the blooms. Members of the class Chrysophyceae were the most dominant taxa in both the March-18 and September-18 samples (Figure 2), accounting for ∼83 and ∼73%, respectively, whilst the other bloom samples only contained ∼4.8% (April-18) and ∼11.9% (March-19). Attempts to resolve the affiliation past the level of class using NCBI Blast indicated that the majority of the OTUs belonged to the order Chromulinales. Despite all the OTUs being affiliated with the class Chrysophyceae, the samples did not share the same OTUs, which would suggest that the species present at the different time points are distinct but from within the same broad taxonomic group. However, further sequencing data that allows resolution to lower taxonomic levels would be required to confirm their precise phylogenetic affiliations. Although members of the green algal class Chlorophyceae were identified in all the samples (Figure 2), they constituted a much smaller percentage of the overall reads. For example, the maximum relative abundance observed in the April-18 sample was only ∼5.0%, whilst the March-18 sample only contained ∼1.6%, where the majority of the OTUs belonged to the order Sphaeropleales. There was another green algal class Mamiellophyceae identified in the sequencing data for the March-18 and April-18 samples, where the relative abundances accounted for ∼0.4% and ∼1.4% of the total reads, respectively, but there were no reads belonging to this class in the September-18 and March-19 samples. The samples also had varying abundances of the diatom class Bacillariophyceae present, with the April-18 and September-18 samples both detected as ∼12%, however, they were less abundant in the other bloom samples. Surprisingly, the sequencing data for the April-18 and March-19 samples indicated that the majority of the reads were not affiliated with algal taxa (Figure 1A), but rather the clade Cryptomycota (∼45.1%, April-18), and the subclass Peritrichia (∼60.1%, March-19), which include ciliated protists. There was a variety of other non-photosynthetic organisms present within the samples, with a greater number of different taxa present in the September-18 and March-19 samples compared to those observed in the March-18 and April-18, albeit mostly at very low relative abundances.

There were a further five samples collected during periods when Chl *a* concentrations were low, four of which exhibited low copy numbers mL^–1^. Furthermore, statistical analysis comparing the copy numbers mL^–1^ of bloom samples and non-bloom samples showed there was a significant difference (one-way ANOVA, *P*-value 0.008, F-crit 4.242), such results confirm the fact that no blooms were evident in the pond when obtaining these samples, as observed by plant operators (Effluent team, Sellafield Ltd, Personal correspondence). The dominant taxa present varied between the samples, for example, members of the class Chrysophyceae were the most abundant organisms identified in the November-18 (∼54.5%), and May-19 (∼37.0%) samples, whilst there was an overwhelming abundance of organisms affiliated with the class Bacillariophyceae in the July-18 sample (∼77.4%) (Figure 2). Despite the high proportions of algal and diatom reads present in these samples, both the qPCR data, and Chl *a* concentrations suggest that the overall microbial load within the pond was insufficient to affect visibility within the water column. The June-18 and August-20 samples were dominated by taxa that were largely absent in the majority of the other samples that were analysed; these were the dinoflagellate order Blastodiniales (∼82%, June-18), and the crustacean subclass Phyllopoda (∼32.2%, August-20).

The June-18 sample was unique in having an estimated copy number mL^–1^ similar to those reported for “bloom” samples, despite low Chl *a* concentrations and an absence of any visible bloom. The dominance of reads affiliated to the dinoflagellate order Blastodiniales is surprising for this sample, given that many members, including the genus *Blastodinium* (Skovgaard et al., 2007, 2012), contain functioning chloroplasts, however, there are some species that are heterotrophic (Stoecker, 1999). Given that there was no visible bloom and pond water Chl *a* concentrations were low during sampling in June-18, it is likely that the dinoflagellates were heterotrophs, although further work is required to determine if this is the case.

Members of the class Chrysophyceae are widely distributed worldwide, and characteristically form blooms in the spring months (Kristiansen, 2009), which may explain their abundance in the PFSP during the spring bloom of 2018. The presence of two unequal flagella are characteristic of these protists (Nicholls and Wujek, 2003), which have a range of modes for nutrient acquisition, and can therefore be heterotrophs, mixotrophs and phototrophs (Bock et al., 2022). A study looking into the nutritional modes of Chrysophyceae found that phototrophic species do not appear to be competitive in alkaline environments, such as those prevalent in the PFSP (pH 9.2-9.5), however mixotrophic organisms are more competitive (Bock et al., 2022). Sequencing of pond water samples taken from the PFSP identified members of the order Chromulinales as the most likely organisms to be inhabiting the pond, which has numerous mixotrophic members such as those belonging to the genus *Chromulina* (Nicholls and Wujek, 2003; Majda et al., 2021). The capacity to switch between different nutrient modes, may provide a competitive advantage to these organisms, enabling them to thrive when nutrients such as phosphate are limited (Kristiansen, 2009; Majda et al., 2021) and may explain their dominance in the 2018 “bloom” samples (March-18 and September-18).

Recently, the microorganisms colonising the First Generation Magnox Storage Pond (FGMSP) at Sellafield, and its associated auxiliary pond were determined (Foster et al., 2020a). Both facilities are outdoor ponds open to the elements. Similar sequencing analysis identified the presence of the class Chrysophyceae in both facilities. During the course of the study, 18S rRNA gene sequencing undertaken on FGMSP water samples, obtained when a bloom was evident, indicated that ∼47.8% of the total reads were affiliated with the class Chrysophyceae. Despite the high proportion of this golden alga grouping in the sequencing data from the FGMSP, analysis of the Chl *a* concentrations, and the average copy number mL^–1^ of the 18S rRNA gene indicated that eukaryotic organisms were unlikely to be forming the bloom. The auxiliary pond, which also had a visible bloom at the time of sampling, supported an overall higher abundance of eukaryotic organisms when compared to the FGMSP, with the highest relative abundance of Chrysophyceae present in May 2016, accounting for ∼30.1% of the total reads obtained (Foster et al., 2020a). The conditions in the FGMSP and the auxiliary pond were different to one another, with the FGMSP maintained at a pH ∼11.4 through the purging of alkaline dosed water and significant levels of radiation present in the pond (1500–2000 Bq mL^–1^, 500–650 μSv h^–1^, ^137^Cs) (Foster et al., 2020a). In contrast, the auxiliary pond had lower pH (9.8–10.9), and lower radiation levels (100–200 Bq mL^–1^, 35–75 μSv h^–1^, ^137^Cs) and did not have a purge system in place (Foster et al., 2020a). Whilst it is not possible to determine if members of the class Chrysophyceae identified in each facility were the same, the presence in all three systems suggests that some members of this class are capable of withstanding significant doses of radiation.

Further work is required, perhaps utilising metagenomics or alternative sequencing platforms that are capable of sequencing larger sections of the 18S rRNA gene, to provide more detailed information about which members of the class Chrysophyceae are present in the pond. Future sampling campaigns would benefit from samples being collected more frequently, and with replicates, while direct measurements of biomass and cell morphology would also be useful to better understand the microbial ecology of the PFSP. However, the acquisition of such samples is not trivial, and the levels of radioactivity associated with these samples presents challenges to carry out even standard measurements.

### 16S rRNA gene analysis

**FIGURE 3:**
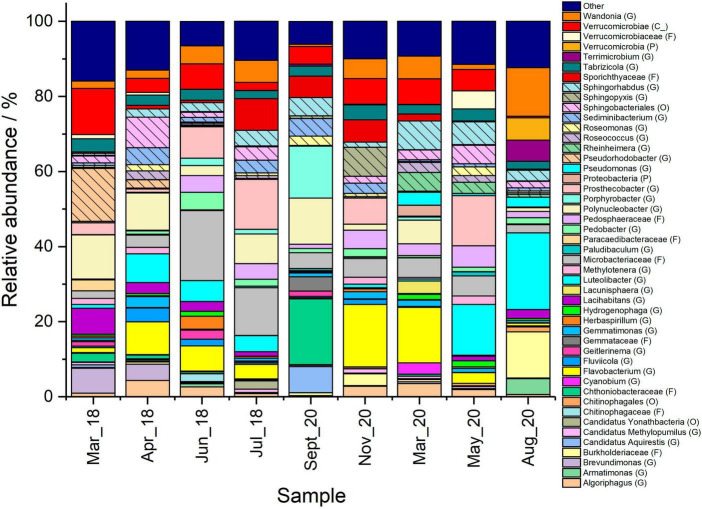
Comparison of prokaryotic organisms in the Pile Fuel Storage Pond between March 2018 and August 2020. Where the relative abundance was 1% or less the data was omitted and placed within the other group. Taxonomic affiliations indicated in parentheses where G, genus; F, family; O, order; C, class; P, phylum.

Sequence analysis of the 16S rRNA genes amplified from the ponds was also carried out to characterise the prokaryotic community present in the samples. The number of OTUs identified in the samples ranged from 98 to 194, with four different phyla that were dominant in all the samples analysed; these were the Proteobacteria, Bacteroidetes, Verrucomicrobia, and Actinobacteria (Figure 3). Proteobacteria were most abundant within the bloom samples, accounting for between ∼40 and ∼53% of the total 16S rRNA gene reads in the samples, whilst non-bloom samples contained between 20 and 28%. Members of the phylum Verrucomicrobia were more heavily represented in the May-19 and August-20 samples, which were collected after the ultrasonic units had been tuned, where the relative abundance increased from 15 to 26% to in excess of 35% (see below).

Of particular interest was the presence of cyanobacterial taxa and other prokaryotic organisms that are known to synthesise bacteriochlorophyll, and therefore, have the potential to acquire energy through photosynthesis. The relative abundance of OTUs affiliated with the Cyanobacteria phylum varied over the course of the sampling campaign, but did not exceed 4% of the total reads in any of the samples (Supplementary Figure 4). Cyanobacteria accounted for less than 1% of the overall reads in the samples where no blooms were reported, with the exception of the June-18 sample, where they accounted for 2.8% of the reads. The samples collected where there were elevated Chl *a* concentrations, and phycocyanin (albeit at much lower levels compared to Chl *a*; Supplementary Table 1), did show slightly higher overall abundance of cyanobacteria and ranged from 0.9% (April-18) to 3.9% (March-19). In addition to cyanobacteria, photosynthetic prokaryotic organisms that have been shown to contain bacteriochlorophyll accounted for ∼2-18% of the overall reads, with the highest abundance present in the September-18 bloom sample, however the qPCR data collected at this time point showed comparable average copy numbers of the 16S rRNA gene to when there was no bloom (Supplementary Table 1). The qPCR results for the 16S rRNA gene indicated prokaryotic organisms were not contributing to the reduction in visibility, and given the low relative abundance of OTUs affiliated with cyanobacterial taxa, it provides further evidence that they were not the main photosynthetic bloom-forming organisms.

### The effectiveness of ultrasonic treatment for controlling microbial growth and bloom formation in the PFSP is potentially increased when refined using taxonomic sequencing data

Prior to the introduction of the ultrasonic units on the 6^th^ April 2018, the PFSP experienced extended periods where microbial growth significantly reduced the visibility in the water, disrupting waste retrieval and pond operations. During the study period the operational settings of the ultrasonic probes were adjusted in an attempt to find a suitable programme to control the microbial population, with settings designed to target either cyanobacteria or green algae selected, until the sequencing data from 2018 was used to change the settings on 8^th^ April 2019 to target flagellates, and remained on this new setting for the remainder of the study period.

The 18S rRNA gene sequencing data collected in 2018, showed the continued presence of Chrysophyceae species which accounted for the majority of the reads in the March-18 and September-18 bloom samples, along with green algae species belonging to the class Chlorophyceae, and diatoms belonging to the class Bacillariophyceae. The sequencing analysis of the samples in this study, did not show any evidence that there was any loss of taxa following the introduction of the ultrasonic treatment or after the adjustment to the settings. The photosynthetic taxa mentioned previously were identified in all the samples analysed, however, since these are broad taxonomic groups, there might be changes in the species present that cannot be deduced from these broad comparisons. As noted above, efforts to sequence larger sections of the 18S rRNA gene, or the use of metagenomics is required to resolve which specific organisms are present in the PFSP, and therefore provide a better understanding of any changes at the lower taxonomic levels over time.

The Chl *a* concentration data collected between 25^th^ January and 6^th^ April each year of the study allows a direct comparison of the PFSP prior to the introduction of the ultrasonic units, while it was operational, and after the settings had been adjusted. In 2018 there were 51 days (71%) where the Chl *a* concentrations were above 10 μg L^–1^, and 38 days (53%) above 20 μg L^–1^. The following year (2019), when the ultrasonic treatment was in place, there was a slight reduction in the number of days above 10 μg L^–1^ Chl *a* (48 days, 66%), and even fewer above 20 μg L^–1^ Chl *a* (17 days, 24%), which suggests while the ultrasonic treatment had not succeeded in preventing the spring bloom there had been a reduction in the number of days where there was a total loss in visibility, i.e., where Chl *a* concentrations were above 20 μg L^–1^. Following the changes made to the ultrasonic units, which were informed by the sequencing data that was collected in 2018, the concentration of Chl *a* did not exceed 10 μg L^–1^ throughout the same period in 2020. The overall Chl *a* concentrations between 25^th^ January and 6^th^ April were compared year on year and revealed significant differences in the concentrations following the introduction of the ultrasonic unit on 6^th^ April 2018 (2018 vs. 2019; one-way ANOVA: *P*-value 3.33E-05, F crit 3.91), and following the adjustments made to the settings (led by the sequencing results) on 8^th^ April 2019 (2019 vs. 2020; one-way ANOVA: *P*-value 2.66E-29, F crit 3.91). These results suggest that the sequencing directed changes made to the ultrasonic units led to more effective treatment and prevented the microorganisms from forming a bloom during this time period.

Comparisons for the Chl *a* data collected during the bloom period that occurred between August and September could only be made following the introduction of the ultrasonic units. In 2018 there was reduced visibility within the PFSP ([Chl *a*] > 10 μg L^–1^) for 29 days (48%), between 1st August and 30^th^ September with 25 days exceeding 20 μg L^–1^ (and a significant loss in visibility). Despite blooms occurring in both 2019 and 2020 in August/September, the number of days above both threshold concentrations declined in 2020 compared to 2019 following the adjustments made to the settings on the ultrasonic units [18 days, 30% (2019), and 11 days, 18% (2020) above 10 μg L^–1^; and 8 days, 13% (2019), and 5 days, 8% (2020) above 20 μg L^–1^]. Furthermore, the comparisons of the overall Chl *a* concentrations observed between 1st August and 30^th^ September 2018 compared to 2019 and 2020 indicated that there were significant differences to the concentrations following the adjustment of the ultrasonic units (2018 vs. 2019; one-way ANOVA: *P*-value 0.001, F crit 3.92; 2018 vs. 2020; one-way ANOVA: *P*-value 1.53E-05, F crit 3.92). Whilst the ultrasonic treatment did not completely prevent the formation of a bloom during this period, even with the changes made to the settings, there was a reduction in the number of days where turbidity caused pond operations to be halted.

Ideally these findings would be supported by relevant control systems, such as systems where (1) no ultrasonic treatment was in place, and (2) ultrasonic treatment was maintained on initial settings and altered to target the dominant organisms within the blooms. However, this was not possible due to the unique characteristics and scale of the nuclear facility in question. Nevertheless, future work on representative microorganisms that were present in the pond on a lab- or meso-scale could provide support to the data collected here, and perhaps provide further evidence that ultrasonic treatment is more effective when informed by sequencing data. Overall, however, the effectiveness of the ultrasonic treatment appeared to be improved when sequencing data was used to inform the settings used (e.g., optimal frequency, amplitude, waveform, and signal duration), and offers potential for the control of microorganisms in challenging radioactive facilities. Future efforts will focus on ensuring that the ultrasonic treatment remains effective, and will include continued monitoring of the microorganisms in the pond through sequencing.

### Microorganisms in other SNFPs

The presence of microorganisms within various SNFPs and other nuclear sites has long been established, with the majority of early studies focussing on the potential of native microorganisms in these facilities to carry out microbial induced corrosion (MIC). The SNFP in Confrentes, Spain is perhaps the best studied system for MIC, where steel coupons were used to determine *in situ* biofilm formation, which showed members of the genera *Burkholderia*, *Bacillus*, *Nocardia*, and *Microbacterium* amongst those present in the biofilms (Sarró et al., 2003, 2005, 2007; Chicote et al., 2004, 2005). Concerns over the presence of microorganisms forming biofilms is due not only to the biofouling of surfaces and inventory within the ponds, but the potential damage to fuel containers and other items stored in the ponds as a result of MIC, leading to fuel degradation and the release of radioactive materials (Barton et al., 2022). Biofilm forming organisms reported in the earlier studies such as those focussing on the Confrentes facility, have also been reported in some of Sellafield’s other outdoor facilities, and are also present in the PFSP, such as members of the genera *Stenotrophomonas*, and *Pseudomonas*. Biofilm formation in the PFSP has not been studied to date. Efforts to determine the microbial community in biofilms could provide useful information about whether there are other organisms that might be useful for bioremediation of radionuclide-containing waters and other applications, as well as add to current understanding of biofouling problems in inhospitable nuclear environments. A range of other indoor SNFPs have been studied more recently (Rivasseau et al., 2013; Tišáková et al., 2013; Karley et al., 2017; Bagwell et al., 2018; Ruiz-Lopez et al., 2020), and in some cases have been shown to host stable microbial communities over extended periods of time, as was observed in an indoor SNFP on the Sellafield site that was dominated by the hydrogen-metabolising genus *Hydrogenophaga* (Ruiz-Lopez et al., 2020).

Studies investigating the presence of eukaryotic organisms in SNFPs are more limited than those for prokaryotes. Studies on indoor SNFPs have infrequently identified eukaryotic organisms, a notable exception being the studies by Rivasseau et al. (2010, 2013, 2016) who isolated a highly radiotolerant green algal species, *Coccomyxa actinabiotis*, from a SNFP in France. More recently, the use of metagenomic sequencing of samples from several areas of the Almirarite Alvaro Alberto Nuclear Power Plant (Angra I) in Rio de Janeiro, Brazil, showed that fungal sequences were the most abundant on the surfaces of the walls within the spent fuel pool and the fuel transfer channel, accounting for ∼94% of the overall community (Silva et al., 2018). Outdoor SNFPs such as the PFSP appear to be much richer in terms of eukaryotic communities, and given their exposure to sunlight, support photosynthetic microorganisms that are prone to form blooms. The Sellafield site has several such outdoor SNFPs which have been studied to determine the presence of both prokaryotic and eukaryotic organisms. The first outdoor SNFP on the Sellafield site to be studied was a circum-neutral pH legacy facility that was shown to support a complex and diverse microbial community (MeGraw et al., 2018). The microbial blooms that formed were dominated by a green microalga most closely related to *Haematococcus pluvialis* (MeGraw et al., 2018), and it was proposed that the production of the red carotenoid astaxanthin might help facilitate this species of algae to colonise the pond, due to the antioxidant properties of the pigment (Wang et al., 2003, 2014; Li et al., 2008; Wada et al., 2013). As previously mentioned a more recent study on the FGMSP and the hydraulically linked auxiliary pond revealed the presence of eukaryotic organisms (Foster et al., 2020a), including members of the golden algae class Chrysophyceae, and the diatom class Bacillariophyceae. Whether such taxa were significant in the microbial blooms in the FGMSP is unlikely due to limited evidence of an increase in eukaryotes in the pond at the time, rather the cyanobacterium *Pseudanabaena* was thought to be the most likely candidate causing blooms. The proliferation of microorganisms in SNFPs that significantly reduce visibility within the pond, appears to be limited to outdoor facilities. Interestingly, whilst there are similarities observed between the different pond systems, even as geographically close as those on the Sellafield site, each SNFP is populated by a distinct community of microorganisms with different organisms having been identified as the key bloom-forming organisms. Their characterisation is potentially important to helping underpin bloom control measures (as suggested in this work), while identifying microorganisms capable of withstanding the inhospitable conditions within the SNFPs also provides potential candidate organisms that could be useful for the bioremediation of aquatic systems; for example, *C. actinabiotis* was shown to be able to accumulate a wide range of radionuclides and fission products (Rivasseau et al., 2013). Furthermore a close relative of the *Pseudanabaena* sp. identified in the FGMSP bloom was also shown to bioaccumulate Sr from the growth medium, via the formation of intracellular SrPO_4_ and SrCO_3_ minerals (Foster et al., 2020b), and more recently Cs accumulation in discrete locations within the cytoplasm have been observed (Zhang et al., 2023). Whether the microorganisms in the PFSP exhibit similar capabilities is currently unknown, but efforts to cultivate organisms from the pond and carry out such experiments would be of interest.

## Conclusion

This study aimed to determine the microorganisms responsible for causing blooms within the PFSP that in previous years have resulted in significant losses in the number of days the facility was operational. Over the course of this study, 18S rRNA gene sequencing suggested that eukaryotic organisms were most likely responsible for the blooms, with the most likely candidates belonging to the golden algae class Chrysophyceae, the green algae class Chlorophyceae, and the diatom class Bacillariophyceae. Furthermore, significant differences in the 18S rRNA gene copy number mL^–1^ in bloom samples compared to non-bloom samples supports their role in the bloom formations. Prokaryotes, whilst present did not appear to be causing changes in the visibility due to higher estimated 16S rRNA gene copy numbers observed where there was no bloom, and no significant difference in the copy number mL^–1^ of the 16S rRNA gene between bloom and non-bloom samples. Efforts to control the proliferation of the microorganisms using ultrasonic treatment indicated some success, with an overall reduction in the number of days lost due to microbial blooms following its installation, and significant reductions in overall Chl *a* concentrations during key bloom periods (January–April and August–September). Furthermore, this study presents interesting preliminary evidence that such treatment efforts can be enhanced by consulting sequencing data to determine the most effective operational settings. More frequent and in-depth analysis is required to provide further support to the findings within this study, however ultrasonic treatment offers a promising tuneable method to control microbial populations in large-scale challenging industrial facilities such as the PFSP.

## Consent for publication

Sellafield Ltd. and the National Nuclear Laboratory have provided consent for the publication of this manuscript.

## Data availability statement

The datasets presented in this study can be found in online repositories. The names of the repository/repositories and accession number(s) can be found below: https://www.ncbi.nlm.nih.gov/, PRJNA1017456.

## Author contributions

LF: Conceptualization, Data curation, Formal analysis, Investigation, Methodology, Writing – original draft. CB: Data curation, Writing – review and editing. SH: Data curation, Writing – review and editing. PJ: Conceptualization, Funding acquisition, Writing – review and editing. JP: Conceptualization, Writing – review and editing. JL: Conceptualization, Funding acquisition, Writing – review and editing.
