# Supplementary material for: Identification of algal rich microbial blooms in the Sellafield Pile Fuel Storage Pond and the application of ultrasonic treatment to control the formation of blooms
Source: Front Microbiol. 2023 Oct 4;14:1261801. doi: 10.3389/fmicb.2023.1261801 (PMC10582928; doi:10.3389/fmicb.2023.1261801)
Supplement: Supplementary file 1 [file Data_Sheet_1.docx]

**Supplementary material**

Supplementary Table 1: Summary of the details of the samples, including pond conditions at the time of sampling, average qPCR copy numbers mL^-1^ of both the 16S and 18S rRNA genes, and the relative abundance of a selection of photosynthetic organisms.

|  | **Sample name** | **Mar_2018** | **Apr_2018** | **Jun_2018** | **Jul_2018** | **Sept_2018** | **Nov_2018** | **Mar_2019** | **May_2019** | **Aug_2020** |
| --- | --- | --- | --- | --- | --- | --- | --- | --- | --- | --- |
|  | Sample date | 07/03/2018 | 12/04/2018 | 19/06/2018 | 10/07/2018 | 06/09/2018 | 25/11/2018 | 25/03/2019 | 27/05/2019 | 26/08/2020 |
| pond conditions | Chlorophyll concentration / µg L¯¹ | 31.81 | 35.80 | 2.03 | 3.32 | 29.83 | 3.03 | 23.79 | 2.52 | 1.28 |
|  | Phycocyanin concentration/ µg L¯¹ | 1.25 | 1.33 | 0.32 | 0.12 | 1.12 | 0.35 | 0.98 | 0.35 | 0.22 |
|  | Temperature/ °C | 3.67 | 7.84 | 16.60 | 20.20 | 16.43 | 8.21 | 8.00 | 14.62 | 17.72 |
| 18S rRNA gene analysis | relative abundance Chrysophyceae | 83.23 | 4.83 | 1.52 | 0.57 | 73.63 | 54.5 | 11.9 | 36.98 | 11.12 |
|  | relative abundance Chlorophyceae | 1.58 | 4.98 | 0.13 | 0.09 | 3.7 | 0.94 | 2.91 | 0.09 | 0.58 |
|  | relative abundance of Mamiellophyceae | 0.39 | 1.43 | 0.2 | 2.36 |  | 0.07 |  |  | 1.71 |
|  | relative abundance of Cryptophyceae | 1.55 | 2.06 | 3.20 | 3.42 | 0.12 | 0.60 |  |  | 2.64 |
|  | relative abundance diatom | 1.16 | 12.17 | 1.39 | 77.45 | 12.36 | 18.09 | 3.78 | 0.01 | 6.95 |
|  | average copy number / mL | 5.96E+04 | 1.77E+04 | 1.14E+04 | 4.16E+03 | 2.25E+04 | 5.33E+03 | 3.40E+04 | 3.91E+03 | 2.84E+03 |
| 16S rRNA gene analysis | relative abundance Cyanobacteria | 2.58 | 0.85 | 2.83 | 0.68 | 1.63 | 0.25 | 3.85 | 0.08 | 0.59 |
|  | relative abundance Chloroflexi | 1.09 | 0.17 | 0.37 | 0.28 | 0.12 | 0.14 |  | 0.07 | 0.02 |
|  | relative abundance photosynthetic bacteria | 3.25 | 9.87 | 4.45 | 5.47 | 18.28 | 2.94 | 6.23 | 6.17 | 2.21 |
|  | average copy number / mL | 1.55E+05 | 1.23E+05 | 1.02E+06 | 4.54E+05 | 4.57E+04 | 3.72E+04 | 3.92E+05 | 3.50E+01 | 3.27E+04 |

Supplementary table 2: Details of the 16S and 18S rRNA gene targets including primer information, and PCR cycling conditions

| Target | Read length | Forward primers | Reverse primers | PCR cycling conditions |
| --- | --- | --- | --- | --- |
| 16S rRNA V4 hyper variable region | 2 × 250-bp paired-end sequencing (Illumina) (Caporaso et al., 2011, 2012) | 515F, 5′-GTGYCAGCMGCCGCGGTAA-3′ | 806R, 5′-GGACTACHVGGGTWTCTAAT-3′ | initial denaturation at 95°C for 2 min, followed by 36 cycles of 95 °C for 30 s, 55 °C for 30 s, 72 °C for 1 min, and a final extension step of 5 min at 72 °C |
| 18S rRNA | 2 × 250-bp paired-end sequencing (Illumina)(Amaral-Zettler et al., 2009) | forward primer, 1391F, 5′-GTACACACCGCCCGTC-3′ | reverse primer, EukBR, 5′-TGATCCTTCTGCAGGTTCACCTAC-3′ | initial denaturation at 95°C for 2 min, followed by 36 cycles of 95°C for 30 s, 57°C for 30 s, 72°C for 1 min, and a final extension step of 5 min at 72°C |

Amaral-Zettler, L. A., McCliment, E. A., Ducklow, H. W., and Huse, S. M. (2009). A method for studying protistan diversity using massively parallel sequencing of V9 hypervariable regions of small-subunit ribosomal RNA Genes. *PLoS One* 4, 1–9. doi:10.1371/journal.pone.0006372.

Caporaso, J. G., Lauber, C. L., Walters, W. A., Berg-Lyons, D., Huntley, J., Fierer, N., et al. (2012). Ultra-high-throughput microbial community analysis on the Illumina HiSeq and MiSeq platforms. *ISME J.* 6, 1621–1624. doi:10.1038/ismej.2012.8.

Caporaso, J. G., Lauber, C. L., Walters, W. A., Berg-Lyons, D., Lozupone, C. A., Turnbaugh, P. J., et al. (2011). Global patterns of 16S rRNA diversity at a depth of millions of sequences per sample. *Proc. Natl. Acad. Sci.* 108, 4516–4522. doi:10.1073/pnas.1000080107.

Supplementary Table 3: Details of the mastermix used for the qPCR runs for both the 16S and 18S rRNA genes, where the primers were specific for the target gene. Volumes represent those used per reaction, which are multiplied by the number of samples and aliquots of 23 µL used per reaction with 2µL of sample DNA or standards added to each well.

| Reagent | Volume / µL |
| --- | --- |
| Roche PCR grade water (Roche diagnostics, Mannheim, Germany) | 9.3 |
| 25 µM forward primer | 0.4 |
| 25 µM reverse primer | 0.4 |
| 1 in 500 diluted ROX reference dye (Aligent, Santa Clara, USA) | 0.4 |
| 2X qPCR SYBR green master mix (Aligent, Santa Clara, USA) | 12.5 |

Supplementary Table 4: Thermal cycling conditions used for the 16S and 18 S rRNA qPCR reactions

| 16S rRNA gene | | | |
| --- | --- | --- | --- |
| Step of qPCR thermal cycling | | Temperature / °C | Duration |
| Initial denaturation | | 94 | 3 min |
| 35 cycles | Denaturation | 94 | 30 s |
|  | Annealing | 50 | 30 s |
|  | Extension | 72 | 60 s |
| 18S rRNA gene | | | |
| Step of qPCR thermal cycling | | Temperature / °C | Duration |
| Initial denaturation | | 94 | 4 min |
| 35 cycles | Denaturation | 94 | 30 s |
|  | Annealing | 55 | 30 s |
|  | Extension | 72 | 45 s |


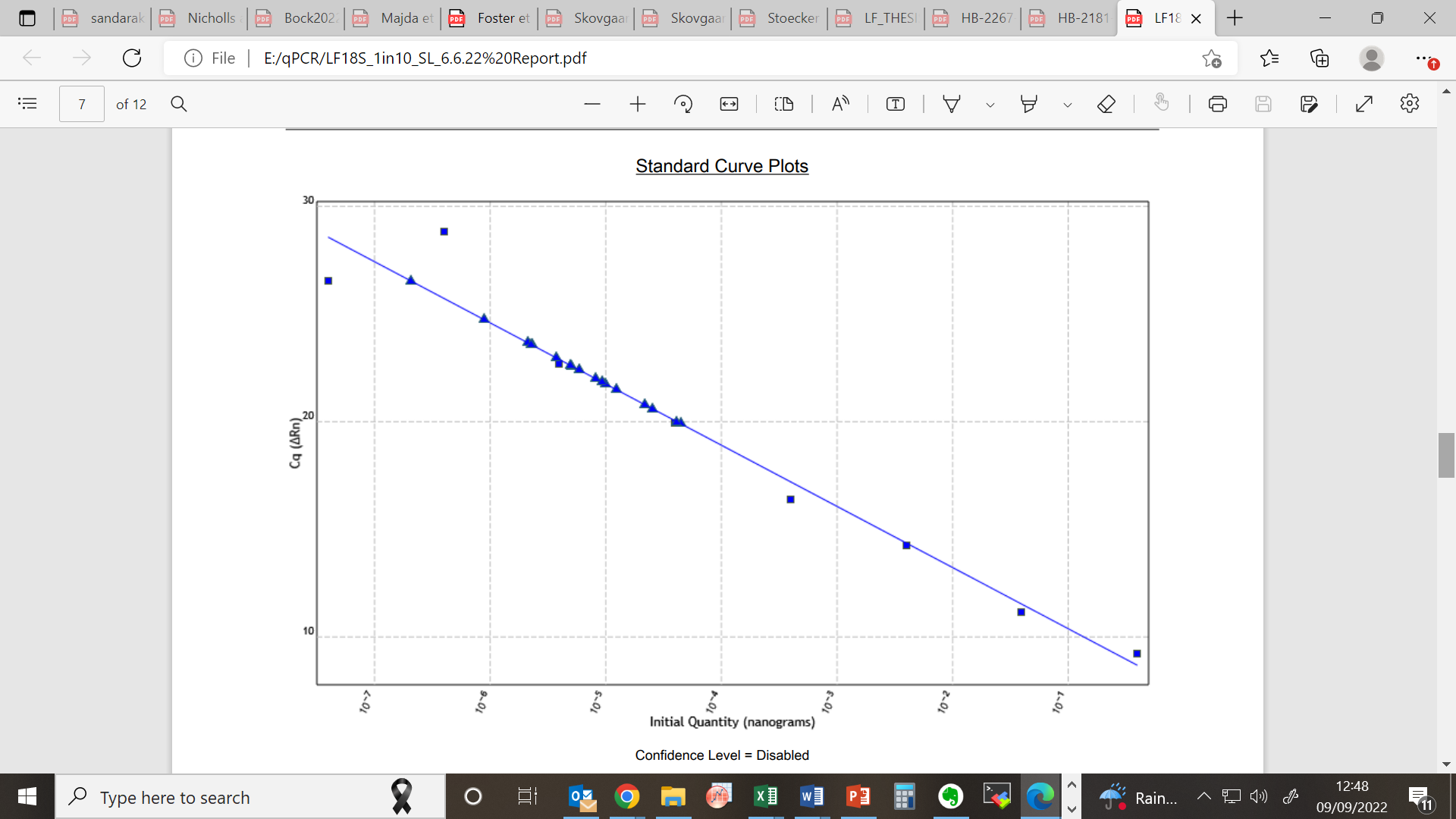


Supplementary Figure 1: Plot of standard curve data for the 18S rRNA gene qPCR run


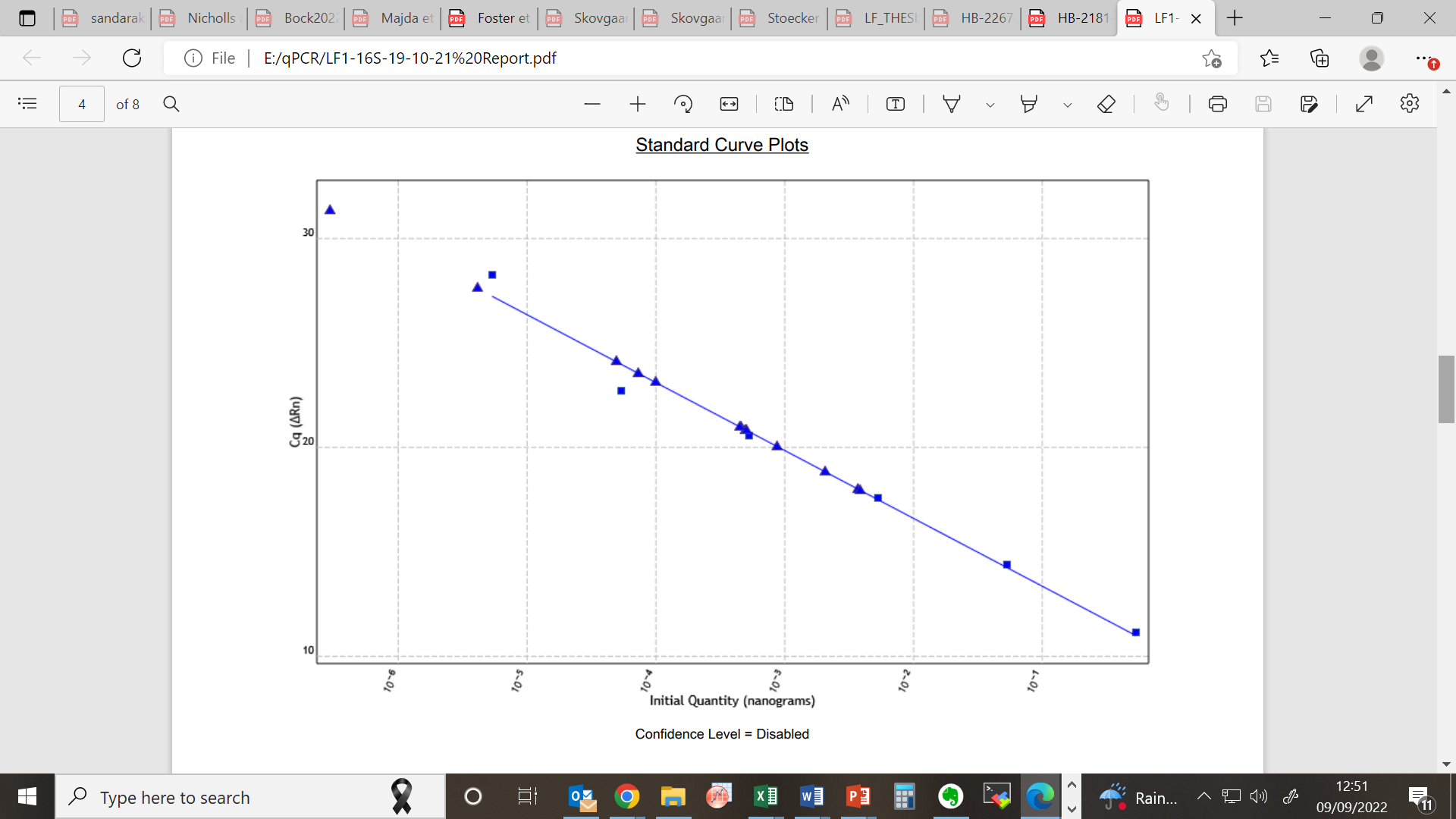


Supplementary Figure 2: Plot of the standard curve data for the 16S rRNA gene qPCR run


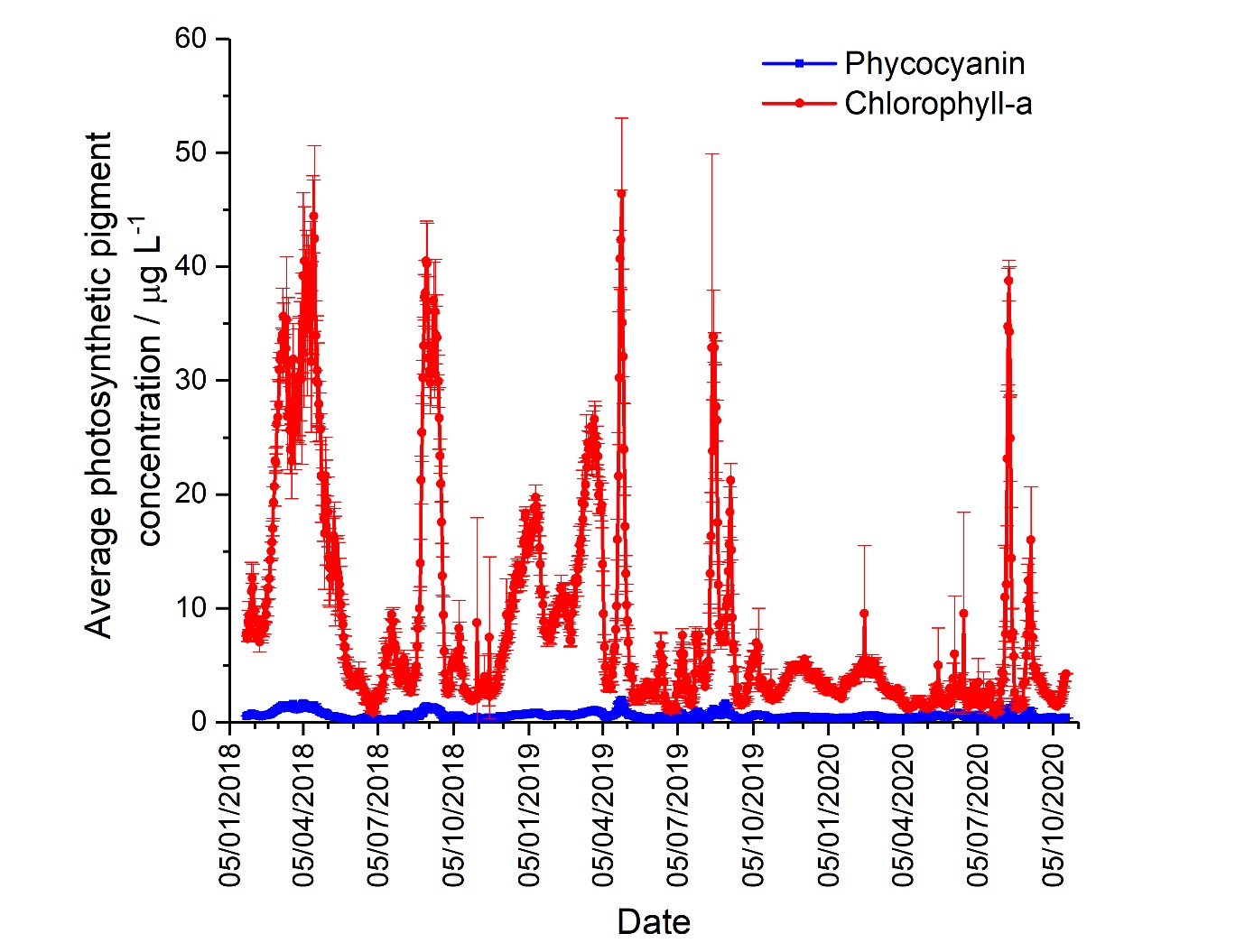


Supplementary Figure 3: Comparison of the average concentration of the photosynthetic pigments phycocyanin (blue) and chlorophyll*a* (red) in µg L^-1^ between 25/01/2018 and 20/10/2020. Error bars denote the standard deviations


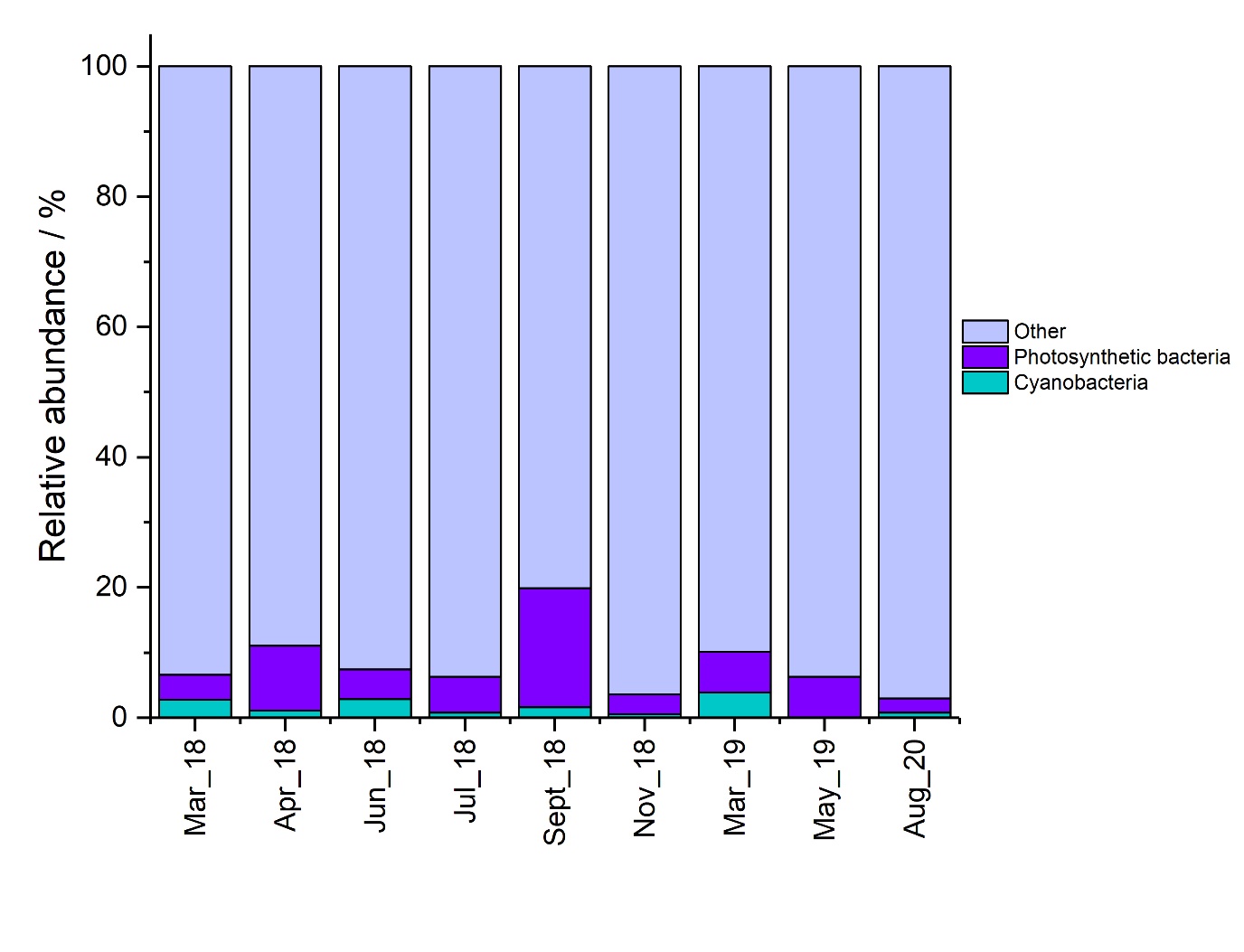


Supplementary Figure 4: grouping of the 16S rRNA gene sequencing data to reflect the broad grouping of cyanobacteria OTUs, prokaryotic organisms that have been shown to contain bacteriochlorophyll*a* and described as photosynthetic bacteria, all other prokaryotic affiliations that do not fall into these categories.
